# Evolution of Mutational Landscape and Tumor Immune-Microenvironment in Liver Oligo-Metastatic Colorectal Cancer

**DOI:** 10.3390/cancers12103073

**Published:** 2020-10-21

**Authors:** Alessandro Ottaiano, Michele Caraglia, Annabella Di Mauro, Gerardo Botti, Angela Lombardi, Jerome Galon, Amalia Luce, Luigi D’Amore, Francesco Perri, Mariachiara Santorsola, Fabienne Hermitte, Giovanni Savarese, Fabiana Tatangelo, Vincenza Granata, Francesco Izzo, Andrea Belli, Stefania Scala, Paolo Delrio, Luisa Circelli, Guglielmo Nasti

**Affiliations:** 1 Department of Abdominal Oncology, SSD-Innovative Therapies for Abdominal Cancers, Istituto Nazionale Tumori di Napoli, IRCCS “G. Pascale”, Via M. Semmola, 80131 Naples, Italy; mariachiara.santorsola@istitutotumori.na.it (M.S.); g.nasti@istitutotumori.na.it (G.N.); 2 Department of Precision Medicine, University of Campania “L. Vanvitelli”, Via L. De Crecchio 7, 80138 Naples, Italy; michele.caraglia@unicampania.it (M.C.); angelalombardi@hotmail.it (A.L.); amalia.luce@unicampania.it (A.L.); 3 Biogem Scarl, Institute of Genetic Research, Laboratory of Precision and Molecular Oncology, 83031 Ariano Irpino, Italy; 4 Department of Pathology, Istituto Nazionale Tumori di Napoli, IRCCS “G. Pascale”, Via M. Semmola, 80131 Naples, Italy; annabella.dimauro@istitutotumori.na.it (A.D.M.); g.botti@istitutotumori.na.it (G.B.); f.tatangelo@istitutotumori.na.it (F.T.); 5 INSERM, Laboratory of Integrative Cancer Immunology, Equipe Labellisée Ligue Contre le Cancer, Sorbonne Université, Université Sorbonne Paris Cité, Université Paris Descartes, Université Paris Diderot, Centre de Recherche des Cordeliers, F-75006 Paris, France; jerome.galon@crc.jussieu.fr; 6 AMES-Centro Polidiagnostico Strumentale, Srl, 80013 Naples, Italy; ced@centroames.it (L.D.); genetica@centroames.it (G.S.); lulacir@libero.it (L.C.); 7 Head and Neck Cancer Medical Oncology Unit, Istituto Nazionale Tumori di Napoli, IRCCS “G. Pascale”, Via M. Semmola, 80131 Naples, Italy; f.perri@istitutotumori.na.it; 8 HalioDx, 13288 Marseille, France; Fabienne.Hermitte@haliodx.com; 9 Department of Radiology, Istituto Nazionale Tumori di Napoli, IRCCS “G. Pascale”, Via M. Semmola, 80131 Naples, Italy; v.granata@istitutotumori.na.it; 10 Hepatic Surgery Unit, Istituto Nazionale Tumori di Napoli, IRCCS “G. Pascale”, Via M. Semmola, 80131 Naples, Italy; f.izzo@istitutotumori.na.it (F.I.); a.belli@istitutotumori.na.it (A.B.); 11 Functional Genomics, Istituto Nazionale Tumori, IRCCS “G. Pascale”, Via M. Semmola, 80131 Naples, Italy; scalaste@gmail.com; 12 Colorectal Abdominal Surgery Division, Istituto Nazionale Tumori, IRCCS “G. Pascale”, Via M. Semmola, 80131 Naples, Italy; p.delrio@istitutotumori.na.it

**Keywords:** colorectal cancer, liver metastases, next generation sequencing, *KRAS*, *SMAD4*

## Abstract

**Simple Summary:**

About 10% of colorectal cancer patients presents with oligo-metastatic disease. The aim of our study was to assess genetic and immunologic dynamics underlying the oligo-metastatic status, evaluating genotype-phenotype correlations in a clean and homogeneous clinical model of liver-limited metastatic colorectal cancer. We show that loss of *KRAS* and *SMAD4* mutations characterizes the oligo-metastatic disease while a progressive mutational evolution (gain in *KRAS*, *PI3KCA*, *BRAF* and *SMAD4*) is observed in poly-metastatic evolving disease. Furthermore, high granzyme-B+ T-cells infiltration is found in oligo-metastatic lesions. This study can support innovative strategies to monitor clinical evolution and to induce regressive genetic trajectories in cancer.

**Abstract:**

Genetic dynamics underlying cancer progression are largely unknown and several genes involved in highly prevalent illnesses (e.g., hypertension, obesity, and diabetes) strongly concur to cancer phenotype heterogeneity. To study genotype-phenotype relationships contributing to the mutational evolution of colorectal cancer (CRC) with a focus on liver metastases, we performed genome profiling on tumor tissues of CRC patients with liver metastatic disease and no co-morbidities. We studied 523 cancer-related genes and tumor-immune microenvironment characteristics in primary and matched metastatic tissues. We observed a loss of *KRAS* and *SMAD4* alterations and a high granzyme-B+ T-cell infiltration when the disease did not progress. Conversely, gain in *KRAS*, *PIK3CA* and *SMAD4* alterations and scarce granzyme-B+ T-cells infiltration were observed when the tumor evolved towards a poly-metastatic spread. These findings provide novel insights into the identification of tumor oligo-metastatic status, indicating that some genes are on a boundary line between these two clinical settings (oligo- vs. poly-metastatic CRC). We speculate that the identification of these genes and modification of their evolution could be a new approach for anti-cancer therapeutic strategies.

## 1. Introduction

Colorectal cancer (CRC) is the second most occurring neoplasia in men and the third in women, and the second leading cause of cancer-related deaths worldwide. Solid epidemiologic data show that in 2018 there were over 1.8 million new cases [1], raising a significant scientific interest [2,3,4,5,6]. Unfortunately, about 30–40% of patients have at diagnosis a metastatic CRC (mCRC), and an additional 30% will develop it later. Liver is the most typical site of distant spread (about 50% of patients), followed by non-regional lymph nodes, lungs, and peritoneum. Bone, brain, and other sites are infrequent targets of metastatic spread [7,8].

Beside typical poly-metastatic CRC (pmCRC), in clinical practice, many advanced patients (about 5–10%) have an oligo-metastatic CRC (omCRC) associated to an indolent and long-term survival course of the disease. Some authors identified oligo-metastases as cancer involving one to three lesions per organ with a maximum tumor diameter smaller than 7 cm [9,10]. Therefore, beside the tumor burden, it has been also considered the “rate of metastases development,” which is low in the oligo-metastatic phenotype [11,12,13]. However, to date, neither the biologic dynamics underlying the oligo-metastatic status nor eventual specific features of poly-metastatic disease are known. The study of gene evolutions during cancer progression is fundamental to generate hypotheses on their role in om- and pmCRC and find phenotype-genotype correlations. However, the eventual presence of co-morbidities can induce changes in cancer genetics, molecular, and clinical outcomes, complicating both epidemiologic and mechanistic studies. In fact, several genes and related products involved in highly prevalent illnesses strongly concur to cancer heterogeneity, i.e., hypertension [14,15,16], diabetes [17], allergies [18,19,20,21] and inflammatory chronic diseases [22,23,24].

OmCRC represents an intriguing model to investigate the mutational evolution of cancer. We have previously reported that CRC developing in a single nodule lung spread, had atypical genomic trajectories (*KRAS* back mutations), scarce T-cell infiltration into primary tumor, and specific gene mutations (common ERBB2 point mutations and very low incidence of SMAD4 alterations) [25].

The present study was conducted in a well-defined omCRC model (Figure S1) of primary CRC with synchronous single liver metastasis. We performed a comprehensive mutational and immunologic analysis of tumor and its micro-environment among patients without recurrence at 3 years follow-up and patients who rapidly developed poly-metastatic disease (Figure S2) in order to explore and generate hypotheses on the genetic trajectories and immunologic dynamics characterizing oligo-metastatic CRC evolution.

## 2. Results

### 2.1. Characteristics of Patients

From 2013 to 2018, 98 patients subjected to concomitant surgical removal of primary CRC and synchronous liver oligo-metastases (≤3 lesions) were screened for inclusion in the present study (Figure S1). At the end of the selection process, six patients (6.1% of our initially screened series) were identified and divided into (A) patients without recurrence at 3-year follow-up (PAT1, PAT2 and PAT3), and (B) patients whose CRC recurred within 1 year (PAT4, PAT5 and PAT6) (Figure S2). Clinico-pathological and personal characteristics of patients are reported in Table 1 and Table 2. At the time of manuscript presentation, all patients belonging to group A were alive without diseases (AWD). These patients represented a clean model of CRC evolving towards oligo-metastatic liver-limited spread. 

### 2.2. Mutational Concordance, Tumor Mutation Burden (TMB), Microsatellite (MSI) Status and Mutational Signatures

**Figure 1:**
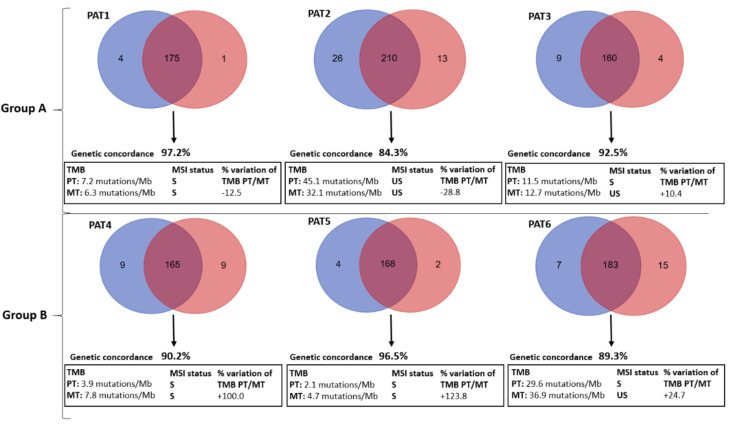
Venn Diagrams with genetic concordance, tumor mutation burden (TMB) and microsatellite instability (MSI) status with percent variation of group A (PAT1, 2 and 3) and group B (PAT4, 5 and 6) patients. Mb: Mega-bas; MT: Metastatic Tumor; PAT: Patient; PT: Primary Tumor. Blue circles represent PT genes’ mutations versus red circles MT genes’ mutations.

A genomic analysis of primary and matched metastatic tissues was performed in order to depict any relevant group-specific genetic trajectory. A descriptive analysis of the overall genetic concordance was done with Venn diagrams; TMB and MSI were also evaluated given their role in the genetic layout of cancer as well as in the neo-antigenic cancer-related load. Figure 1 reports Venn diagrams, TMB, and MSI of enrolled patients. 

**Figure 2:**
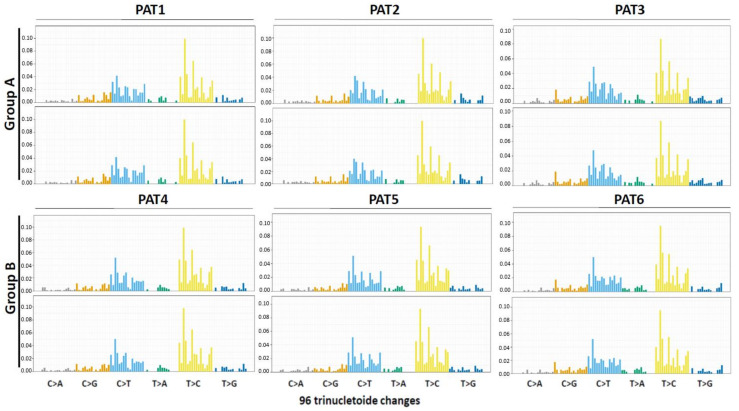
Mutational signatures (see Methods) in group A (PAT1, 2 and 3) and group B (PAT4, 5 and 6) patients. PAT: Patient. The upper panels refer to the primary tumors, the lower panels to the matched metastatic tumors.

The genetic sharing between primary tumors (PTs) and matched liver metastases (Metastatic Tumors: MTs) was high in all cases (from 84.3% in PAT2 to 97.2% in PAT1, mean: 91.7%). Overall, TMB ranged from 2.1 mutations/Mb (PT of PAT5, group B) to 45.1 mutations/Mb (PT of PAT2, group A). TMB variation {[(TMB of MT-TMB of PT)/TMB of PT] × 100} was calculated being a marker of tumor mutational evolution. It ranged from −28.8% to +19.1% in group A vs. +24.7% to +123.8% in group B (*p* = 0.0435). The MSI status was more frequently unstable (three out of six samples) in group A compared to group B (only one sample out of six). The set of somatic mutations observed in a specific cancer, with its distinct patterns of base-pair substitutions (C:G > A:T, T:A > G:C, etc.) found in each trinucleotide context, reflects the mutational processes active during its life history. This represents a fingerprint documenting an aspect of heterogeneity and mutational evolution of cancer. Interestingly, none of the patients had significant PT/MT differences in terms of mutational profiles (>10% of the 96 combinations with delta-frequencies (MT-PT) of specific base-pair mutation types >25%) (Figure 2).

### 2.3. Overall Genes Evolution

**Figure 3:**
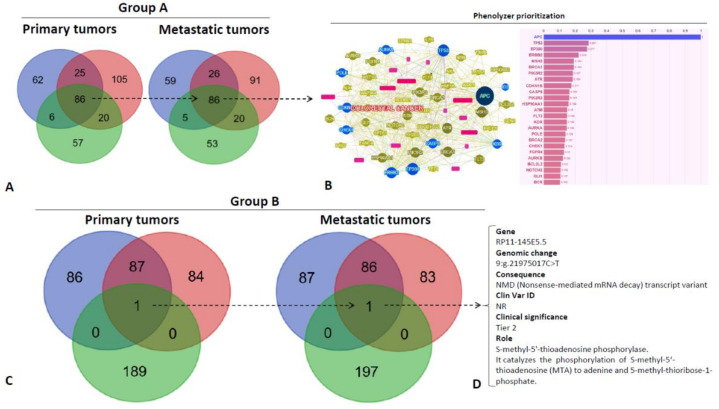
Venn Diagrams of variants shared by primary tumors and metastatic tumors according to group A (**A**) (PAT1, 2 and 3) and group B patients (**C**) (PAT4, 5 and 6). The circles blue refer to genes’ mutations’ of PAT1, the circle red of PAT2 and the circle green of PAT3. Phenolyzer genetic variants prioritization according to genes shared in group A patients (see Methods) (**B**). Only one gene (indicated by the discontinuous arrow is shared in group B and it is emphasized in on the right side (**D**).

The overall gene evolution of groups A and B was also collectively analyzed to find any genotype/phenotype correlations. Venn diagrams were depicted in order to show the extent of shared and private events among PTs and MTs (Figure 3A,C). Interestingly, in group A, 86 genes were shared among PTs, and MTs. Conversely, in group B, only one gene was shared and it was the same in PTs and MTs (Figure 3D) (*RP11-145E5.5*, 9: g.21975017C>T leading to a NMD transcript variant, Tier 2 according to according to AMP/ACMG prioritization of variants). Genetic results of group A were submitted to Phenolyzer to identify any relationships between “seed” genetic variants and “secondary” ones (see Methods). The most dominant and interrelated genes were *APC*, *EP300* and *TP53* (Figure 3B).

### 2.4. Key Driver Genes Evolution

The association between mutations of specific genes and CRC development has been demonstrated by several studies. The landscape of these “driver mutations”, in a case-matched manner (PT vs. MT), is reported in Table 3. At this regard, group A and group B present divergent mutational directions towards the metastatic tumor development. PAT1 appears genetically stable, PAT2 loses alterations of *KRAS* and *SMAD4*, and PAT3 loses a pathogenic mutation of *KRAS*. By contrast, in group B, PAT4 gains mutations in *KRAS* and *PIK3CA*, PAT5 gains mutations in *PIK3CA* and *SMAD4*, and PAT6 progresses in *PIK3CA* and *BRAF* alterations (non-canonical mutation). A complete list of genes’ alterations gained by liver metastases in each patient, along with their role in cancer, AMP/ACMG prioritization and ClinVar ID, is reported in Table S1.

### 2.5. Tumor Microenvironment Evolution

**Figure 4:**
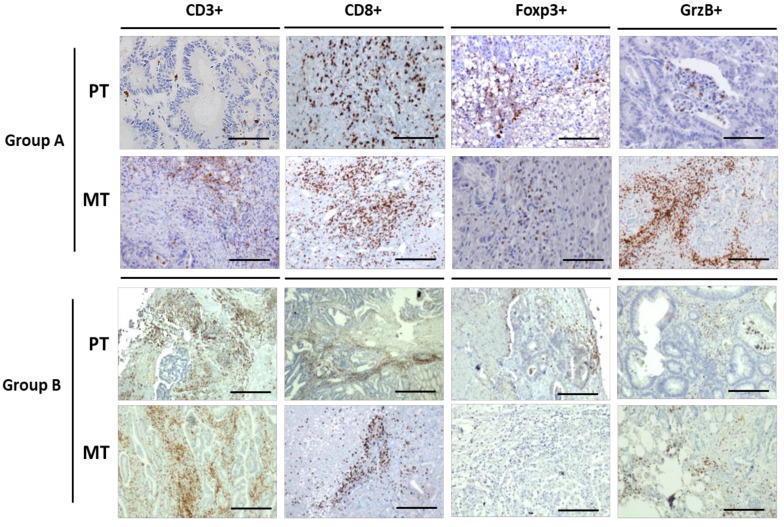
Representative immunohistochemistry of CD3, CD8, Foxp3 and GrzB+ cells (bar = 200 µm).

It is increasingly clear that tumor immune microenvironment has strong influence on tumor initiation and progression in CRC. Therefore, to analyze the dynamics of the immunological microenvironment, CD3+, CD8+, FoxP3+, and GrzB+ cells were quantified in matched PT and MT tissues by IHC. The density of each cell subset (means ± 2SD) is reported in Table 4. Three random cases were also submitted to immunoscore assessment. Immunoscore represents the most objective and complex evaluation of tumor immunological microenvironment integrating digital pathology and a dedicated image-analysis software. Our results were concordant with immunoscore assessments. Densities of T-cell subsets in primary tumors were much more variable than those in the metastatic ones. Significant differences between groups were registered only in CD8+ at invasive margins (IM) of PTs (group A vs. group B: 166.7 ± 16.6 vs. 533.3 ± 88.1; *p* = 0.0150) and in GrzB+ cells into tumor cores (TC) of MTs (group A vs. group B: 58.6 + 8.6 vs. 27.6 + 2.7; *p* = 0.0270). Considering the high biologic relevance of GrzB+ lymphocytes that are the fully differentiated and activated anti-tumor lytic cells, representative immunohistochemical stainings of group A and group B TCs’ microenvironment for CD3+, CD8+, FoxP3+ and GrzB+ cells is reported in Figure 4.

## 3. Discussion

In the present study we show, for the first time in a well-defined model of CRC, that oligo-metastatic disease follows mutational directions divergent from those of poly-metastatic disease. Differently from our previous report [25] where two out four patients received chemotherapy before surgical metastasis removal, in the present one there was no interference of systemic treatments because patients were subjected at the same time to primary and metastatic tumor surgical removal. The latter condition determined the clear definition of our clinical and pathological model and it could be responsible for the high concordance observed in both mutational signatures and genetic features between PTs and MTs.

Metastases in group A patients slip along a milder phenotypic trajectory characterized by “back” mutations of key driver genes. In fact, in two patients we observed that phenomenon in *KRAS* and *SMAD4*, two conserved genes in previous high-throughput analyses of matched primary-pmCRCs (Table S2). These data were repeated and confirmed through PEPP re-sampling and *KRAS* mutation PCR-based testing. In another good prognosis patient (PAT1) the mutational evolution was stable. Moreover, group A patients presented a high GrzB+ T cell-subsets in tumor core of MTs microenvironment revealing enhanced activation of tumor-infiltrating lymphocytes (TILs) in those lesions (where two out of three were MSI unstable).

Conversely, in group B, the mutational directions were characterized by “forward” mutations in key driver genes in all cases (*KRAS* and PIK3CA in PAT4, *PI3KCA* and *SMAD4* in PAT5, and *PIK3CA* and *BRAF* in PAT6) and tumor immune microenvironment was infiltrated by a significantly low density of GrzB+ CD8+ cells. Therefore, despite a similar clinical presentation, the cancer population in group B evolved along both progressive oncogenic mutations in CRC development and immunological escape.

The evolution of some specific genes deserves discussion given their known or putative role in CRC. In fact, *PIK3CA* mutations were gained in all MTs of patients with poor prognosis. Phosphatidylinositol 3-kinases (PI3Ks) are lipid kinases that phosphorylate phosphoinositidies at the D3 position of the inositol and produce intracellular messengers regulating proliferation, adhesion, and migration. Mutations in the p110alpha subunit (*PIK3CA*) are associated with anti-cancer drug resistance, aggressive histologic features, and poor overall survival [26,27]. Similar effects, as already described above, (forward mutations) were observed for *KRAS* (PAT4), *NRAS* and *SMAD4* (PAT5) and *BRAF* (PAT6).

In total, 86 genes were shared among PTs and MTs in group A. Phenolyzer tool was applied in order to evidence the most dominant and interrelated genes. Interestingly, along *APC* and *TP53*, which have a defined role in CRC [28], we found *EP300* gene among the first three dominant ones. *EP300* gene encodes a histone acetyl-transferase (HAT p300) involved in the epigenetic regulation of chromatin activity [29]. This transcript can influence important cell processes such as proliferation and differentiation. *EP300* mutations have been found in many cancers including CRC; however, its precise role in tumorigenesis is debated and contradictory, since some studies showed a tumor suppressor function, while others showed oncogenic co-activation properties [30,31]. Our results indicate that additional studies are needed to define the role of *EP300* and its relationships with the oligo-metastatic behavior. Interestingly, beside genes involved in proliferation, apoptosis, differentiation, and neoangiogenesis (*ERBB2, PIK3R2* and *3*, *CDKN1B*, *CASP8*, *HSP90AA1*, *FLT3*, *KDR*, *AURKA* and *B*, *FGFR4*, *BCL2L2*, *NOTCH3*, *BCR*), a significant number of other genes evidenced with Phenolyzer tool in group A were involved in DNA repair mechanisms (*MSH3*, *BRCA1*, *ATM*, *POLE*, *BRCA2*, *CHEK1*, *GLI1*). The latter, together with the increased MSI and TMB, could account for high immunogenicity of group A metastases characterized by high density of GrzB+ CD8+ T-cells into tumor cores.

Conversely, in group B patients, we observed a more marked mutational divergence with only shared one gene: *RP11-145E5.5*. Its product is a S-methyl-5’-thioadenosine phosphorylase (MTAP) involved in polyamine biosynthesis. Loss of MTAP activity has been hypothesized to play a role in malignant melanoma [32,33]. Unfortunately, very little is known in CRC where it appears overexpressed compared to normal mucosa and positively related to aggressiveness of CRC cells [34,35]. Another important ancillary observation of our study is the alterations of some genes correlated to the homing of metastases to the liver. In details, the following genes were frequently altered in PTs and MTs of our series: *HSP90AA1, NR4A2, KDR, FLT3* and *RPS6KB2* [36,37]. Notably, they can act directly by promoting cell migration, EMT promotion and proliferation (*KDR, FLT3*) or indirectly through pleiotropic actions (HSP90AA1: protein stabilization; NR4A3: epigenetic modifications; RPS6KB2: protein synthesis). Even though such aspects are beyond the scope of this work, additional studies are needed to verify the effects of these genes (*HSP90AA1, NR4A3, RPS6KB2*) on the expression of proteins involved in CRC metastatization (chemokine receptors, adhesion molecules, growth factors, etc.).

The strength of our work resides on the rigorous effort we made to select patients having cancer as the only illness. In fact, as previously discussed, many prevalent diseases may have a profound impact on cancer heterogeneity and interfere through common genetic mediators. Previous published analyses, even if larger than the present one, suffered from extreme heterogeneity (different stages, heterogeneous and sequential treatments, different comorbidities, etc.) and this certainly affects results’ interpretation. Therefore, although on a smaller series of patients, this work contains a strong hypothesis-generating power on the different mutational directions of om- vs. pm-CRC.

Liquid biopsy, whose dissertation is beyond the scope of this work, although still not a standard procedure, could represent a very useful tool to monitor tumor mutational evolution in CRC [37]. Interestingly, we have observed disappearance of *KRAS* mutations through liquid biopsies in pmCRC (manuscript in preparation). This phenomenon was much more frequent in specific pathogenic mutations (*p*.G12D, *p*.G12V, *p*.G13D) and it was associated with a better prognosis. Furthermore, we have recently shown, in a very clean model of oligo-metastatic CRC, that regressive trajectories of specific “backbone” mutations (as *SMAD4* and *KRAS*) can associate with a better clinical course [25].

Some limits deserve to be evidenced and discussed. First, we cannot rule out the hypothesis that back mutations occurred spontaneously, lacking direct evidences that group A neoplastic mutated clones were specifically recognized and eliminated by GrzB/CD8+ T-cells (immune mediated sub-clonal selection). Second, the size of our work was necessarily limited by the very high selection we made. In fact, considering all patients submitted to hepatic surgery for mCRC in the analyzed period (2013–2018) in our institute, only 1.3% (6/444) had the clinical characteristics to be included in this study. Third, we did not analyze tumor tissues from group B patients at progression because of unavailability of subsequent tissues (further surgery or solid/liquid biopsies). This was a “missed opportunity” in those patients to record mutational and immunologic dynamics of poly-metastatic spread. We have also to discuss that the negative prognosis of group B could be influenced by, at least, two clinico-pathological factors of primary tumors: (i) the lymph node involvement (all patients were N+) and (ii) the sidedness (two patients had right-sided tumors). However, only PAT5 had N2b involvement, while PAT4 and 6 had only two positive lymph-nodes, and the 5-year survival of these patients is quite similar to stage II (about 60%). Moreover, the right-sided CRC prognosis is related to the biology and initial stage of disease rather than to the tumor site itself. On the other hand, all cases of our series presented with stage IV disease due to the presence of single liver metastases. In this clinical setting, oncologists are always called up to plan the same therapeutic strategy, often based on adjuvant-like administration of chemotherapy.

## 4. Materials and Methods

### 4.1. Tumor Specimens and Sequencing

Matched Formalin-Fixed and Paraffin-Embedded (FFPE) tissue specimens of primary colorectal cancer and single-nodule liver metastases were collected following a strict selection (Figure S1). The study was approved by the Scientific Director of our Institute (Prof.Gerardo Botti, Scientific Director of the Istituto Nazionale Tumori di Napoli, IRCCS “G. Pascale”, via M. Semmola, 80131, Naples, Italy). All patients admitted in our Institution sign an informed consent to allow any biologic studies on their peripheral blood cells and/or fresh or FFPE tissues. Ten *μ*M serial sections were cut from each tissue specimen for microdissection of tumor cells under morphological control. DNA isolation was performed through the MGF03-Genomic DNA FFPE One-Step Kit, according to the manufacturer’s protocol (MagCoreDiatech, RBCBioscience, Ln. 235, Baoqiao Rd., Xindian Dist., New Taipei City 23145, Taiwan). DNA quality was established in triplicate using the FFPE QC Kit according to the manifacturer’s protocol (Illumina, San Diego, CA, USA). Libraries were prepared with TruSigtTMOncology 500 kit, based on target enrichment that analyzes 523 cancer-relevant genes. The assay detects small nucleotide variants (SNVs), indels, splice variants, and immunotherapy biomarkers such as tumor mutational burden (TMB) and microsatellite instability (MSI) (see below). Sequencing was performed on an Illumina NovaSeq 6000 (San Diego, CA, USA) platform.

### 4.2. Tumor Mutational Burden (TMB), Microsatellite Instability (MSI) and Mutational Profiles

TMB was measured by exome sequencing [38], counting all coding, somatic base substitutions and indels in the targeted regions, including synonymous alterations. The size of the targeted (coding) genomic region was 1.9 Mb. MSI as result of impaired DNA mismatch repair represents a phenotype of clinical significance in colorectal cancers. A highly accurate exome-based predictive model for the MSI phenotype was used, it resides on a statistical MSI classifier from somatic mutation profiles that separates MSI-H (MSI-high) from MSS (MS stable) tumors [39]. The MSI classifier was trained using 999 exome-sequenced TCGA tumor samples with known MSI status (i.e., assayed from mononucleotide markers), and obtained a positive predictive value of 98.9% and a negative predictive value of 98.8% on an independent test set of 427 samples.

The set of somatic mutations observed in a cancer and distinct patterns of substitution types reflect the specific mutational processes that have been active during its life history. Here, with a descriptive aim, we reported the matched mutational profiles of primary and metastatic lesions displaying the fraction of mutations found in each trinucleotide context. A mutational signature is the combination of the frequencies of all base-pair mutation types (C:G  >  A:T, T:A  >  G:C, etc.) and their flanking nucleotides; the convention is to annotate mutations from the pyrimidine (C  >  A, T  >  A, etc.). The figures plot all 96 possible combinations of mutation types and neighboring bases.

### 4.3. Tumor-Infiltrating Lymphocytes Analysis

Analysis of T cell subsets in tumor microenvironment was conducted through immunohistochemistry (IHC). Formalin-fixed, paraffin-embedded 4-μm tissue sections of primary CRC and liver metastases were immunostained according to a biotin-streptavidin-peroxidase method (YLEM kit, Rome, Italy). Before incubation with primary antibodies, sections were subjected to (i) routine deparaffinization, (ii) rehydration, (iii) treatment with Dako target retrieval solution, (iv) incubated for 10 min on a hot plate (95–99 °C), (v) allowed to cool for 20 min, (vi) incubated for 10 min in 3% hydrogen peroxide in distilled water, (vii) washed in PBS thrice for 5 min, (viii) incubated with 10% normal horse serum in PBS for 30 min, and (ix) washed with PBS buffer. Treatment with primary antibodies (anti-human CD3, anti-human CD8, anti-human FoxP3, and anti-human Granzyme B [40]) was done for 2 h at room temperature. The antigens were then revealed through a biotin-labeled secondary antibody/streptavidin-peroxidase/diaminobenzidinetetrahydrochloride method: sections were incubated with biotin-labeled secondary antibody (1:30), streptavidin-peroxidase (1:30) for 20 min each, and stained for 5 min with 0.05% 3,3′-diaminobenzidine tetrahydrochloride freshly prepared in 0.05 mol/L Tris-HCl buffer (pH 7.6) containing 0.024% hydrogen peroxidase. Finally, slides were counterstained with hematoxylin, dehydrated, and mounted in Diatex. Negative controls were obtained by substituting the primary antibody with a mouse myeloma protein of the same subclass at the same concentration as the monoclonal antibody. Slides were scanned through an automated scanning microscope and image analysis system (Genetix, San Jose, CA, USA). Cell density was expressed as cells/mm^2^. All qualitative and quantitative analyses of T-cell subsets were reviewed by two pathologists blinded to all clinical information. The tumor microenvironment was morphologically divided into tumor core (TC) and invasive margins (IM) as previously described [41].

### 4.4. Biostatistical Analysis and Data Presentation

Illumina TruSigth Oncology 500 bioinformatics pipeline was applied to analyze sequencing results. A median of 92 million reads were generated for each sample and the coverage in the target region was above manufacturer’s suggested threshold of 150×. Sequence data were aligned to the human reference genome GRCh37 (http://www.ncbi.nlm.nih.gov/projects/genome/assembly/grc/human/index.shtml) using the Burrows-Wheeler Aligner with default parameters [42]. Both population- and cancer-specific variants were intersected with GENCODE, dbNSFP, ICGC-PCAWG, COSMIC, 1000Genomes, ClinVar, CancerMine, OncoScore, CIViC, and CBMDB databases to assess the clinical significance of the found mutations. Variants were filtered with unmatched normal datasets and removed if the global minor allele frequency was <1%. The prioritization of variants was done according to a four-tiered structure, adopting the joint consensus recommendation by AMP/ACMG [43]. Variants of strong clinical significance in cancer were defined considering items with the strongest evidence levels in the database for (i) clinical interpretations of variants in cancer (CIViC, civicdb.org), and (ii) Cancer Biomarkers (cancergenomeinterpreter.org/biomarkers). Results about variants shared between PTs and metastatic tumors (MTs) are shown with ID according to ClinVar; however, variants were also manually curated to exclude residual false positives. The scenario in which interpretation for the clinical consequence of a variant was “benign”, but the review status quality was scored <2/4 or reporting conflicting results, was defined as “probably benign”. The TP53 *p*.Pro72Arg variant was reported in this study as potentially relevant for CRC. In fact, while the codon 72 SNPs have limited impact on cancer risk for WT P53, many different research groups showed independently that this SNP markedly influences the activity of tumor-derived mutant forms of p53 (TP53) both in vitro and in vivo [44,45,46,47,48,49]. Complete mutational results can be accessed upon a signed justified request sent to a.ottaiano@istitutotumori.na.it.

The genetic sharing was indicated as the percent of mutational concordance in matched PTs and derivative MTs in all coding variations. Venn Diagrams were depicted in order to plot intersections among genetic results in selected subgroups, and Phenolyzer was used to evidence relevance and relationships between any “seed” genetic variants and “secondary” ones.

Phenolyzer is a computational tool that prioritizes genes on the basis on updated existing knowledge (protein–protein interactions, sharing of biological pathways or gene family, gene–gene transcriptional regulation, etc.). It integrates OMIM, Orphanet, ClinVar, Gene Reviews and GWAS Catalog as gene-disease databases. However, for a complete methodology description see Yang et al. [50]. Results are expressed through a score system and a network visualization tool that integrates gene-gene and gene-cancer relationships providing readers with a panoramic view of the interactional context.

The Disease-Free Interval (DFI) was measured in months and it represented the time elapsed from the surgical removal of PTs to the occurrence of poly-metastatic disease.

Densities of T-cell subsets were expressed as cells/mm^2^ and results represented with the arithmetic averages +2 standard deviations (SD). Cells were also manually counted by two Pathologists six times each. Independent sample t-test was applied to compare means of TMB and T-cell densities between group A and group B assuming samples’ independence. The hypothesis tested was that the difference between the means of groups A and B were equal to 0 (null hypothesis). With a two-tailed probability (*p*) value less than 0.05, the conclusion was that the two means do indeed differ significantly.

## 5. Conclusions

A speculative and hypothesis-generating model, integrated with host/tumor immunologic interaction, about the divergence between om- and pmCRC is reported in Figure S3. Specific progressive oncogenic mutations could represent an evolutionary gain for primary tumors allowing them to develop and to spread towards to new organs. These mutations could represent the boundary line between om- and pm-CRC.

Our findings could open new scenarios on both diagnostic and therapeutic point of views. In fact, we have defined the molecular and genetic bases to define the “true” omCRC setting and planning adjuvant treatments only when the disease shows progressive mutational evolution. Moreover, new studies are needed to understand the molecular bases of the evolution of metastatic clones and its interaction with microenvironment (including immune system and vascular compartment). In fact, the data arising from the present manuscript suggest that a negative immunologic selection of clones with poly-metastatic potential, rather than a spontaneous genetic regression, could account for the clinical oligo-metastatic behavior. Based on these, we are now working to identify these tumor reactive clones in long-surviving patients. The present results suggest also the design of novel strategies for the triggering of regressive trajectories in cancer cells based on the use of adoptive cellular immunotherapy or transcriptional and post-transcriptional gene engineering.

## Figures and Tables

**Table 1:** Clinico-pathological characteristics of the enrolled patients (I).

Patient ID	Year of Diagnosis	Age	Ge	BMI (Kg/m^2^)	Histology, Pathologic Stage at Diagnosis *; Grading (G) in PT and MT	Tumor Side, and Type of Surgery	Ad CT	Sites of Metastases Presentation	TTM (m)
Group A									
PAT1	2014 April	76	F	19.5	Adenocarcinoma, pT3pN1aM1a pV0pR0; G2, G2	Left, combined left hemicolectomy and segment 3 pR0 metastasectomy.	No	None	NR
PAT2	2017 March	60	M	19.8	Adenocarcinoma, pT3pN0M1a pV1pR0; G2, G3	Right, combined right hemicolectomy and segment 5 pR0 metastasectomy.	Capox	None	NR
PAT3	2017 January	81	M	21.0	Adenocarcinoma, pT2pN1bM1apV0pR0; G2, G3	Left, combined left hemicolectomy and segment 6 pR0 metastasectomy.	Cape	None	NR
Group B									
PAT4	2014 October	81	M	18.2	Adenocarcinoma, pT3pN1bM1a pV1pR0; G2, G3	Right, combined right hemicolectomy and segment 5 pR0 metastasectomy.	Cape	Liver, peritoneum	6.0
PAT5	2018 March	61	F	19.0	Adenocarcinoma, pT2pN2bM1a pV1pR0; G2, G2	Right, combined right hemicolectomy and segment 8 pR0 metastasectomy.	Capox	Liver, lungs, peritoneum	3.5
PAT6	2013 March	27	M	18.9	Adenocarcinoma, pT3pN1bM1a pV1pR0; G2, G3	Left, combined left hemicolectomy and segment 8 pR0 metastasectomy.	Capox	Liver	7.5

Ad: Adjuvant; BMI: Body Mass Index; CAPOX: Capecitabine day 1 to 14 every 21 days, and Oxaliplatin day 1 every 21 days; Cape: Capecitabine day 1 to 14 every 21 days; Ge: gender; G: grading; m: months; MT: Metastatic Tumor; NR: Not Reached; pR0: no residual disease at microscopic examination after surgery; pV0/pV1: absence or presence of vascular invasion in tumor specimen; PAT: Patient; PT: Primary Tumor; TTM: Time-To-Metastatization. * according to AJCC (American Joint Committee on Cancer) 8th edition.

**Table 2:** Clinico-pathological characteristics of the enrolled patients (II).

Patient ID	First-Line CT	PFS1 (m)	No. of Cycles; Duration of CT (Months)	Second-Line CT	PFS2 (m)	No. of Cycles; Duration of CT (Months)	Third-Line CT	PFS3 (m)	No. of Cycles; Duration of CT (m)	Vital Status	Survival Time from Diagnosis (m)
Group A											
PAT1	None	0.0	0; 0.0	None	0.0	0; 0.0	None	0.0	0; 0.0	AWD	NR
PAT2	None	0.0	0; 0.0	None	0.0	0; 0.0	None	0.0	0; 0.0	AWD	NR
PAT3	None	0.0	0; 0.0	None	0.0	0; 0.0	None	0.0	0; 0.0	AWD	NR
Group B											
PAT4	FU/Beva	11.0	20; 10.5	Rego	5.5	5; 5.0	None	0.0	0; 0.0	D	18.0
PAT5	Folfox/Beva	3.0	6; 3.0	Folfiri	2.0	4; 2.0	None	0.0	0; 0.0	D	5.5
PAT6	Folfiri/Pani	9.5	17; 9.0	Folfox/Beva	9.0	16; 8.0	Folfiri Rech	2.0	2; 4.0	D	21.0

AWD: Alive Without Disease; Beva: Bevacizumab day 1 every 14 days; D: dead; FU: FloroUracil; Folfiri: Fluorouracil plus Irinotecan day 1 every 14 days; Folfox: Fluouracil plus oxaliplatin day 1 every 14 days; NR: Not Reached; Pani: Panitumumab day 1 every 14 days; PAT: Patient; PFS: Progression-Free Survival; Rech: PT: Primary Tumor; Rechallenge.

**Table 3:** Summary of patient-by-patient CRC key driver mutated genes (Tier1/2/3 according to four-tiered structure of AMP/ACMG consensus).

Groups	Patient	PT Mutated Genes	MT Mutated Genes
Group A	PAT1	APC (p.R554TER), TP53 (p.R273H, p.P72R*), BRCA2 (p.Y1655TER).	*APC (p.R554TER), TP53 (p.P72R, p.R273H), BRCA2 (p.Y1655TER).*
	PAT2	*APC (p.1450TER), TP53 (p.K382TER, p.P72R), KRAS (p.A146T), SMAD4 (p.R361H).*	*APC (p.1450TER), TP53 (p.K382TER, p.P72R).*
	PAT3	*APC (p.S587TER), TP53 (p.V197E, p.V173A, p.P72R), KRAS (p.G13D), KRAS (p.G12D).*	*APC (p.S587TER), TP53 (p.V197E, p.V173A, p.P72R), KRAS (p.G13D).*
Group B	PAT4	*APC (p.Q236TER), TP53 (p.C135F, p.P72R).*	*APC (p.Q236TER), TP53 (p.C135F, p.P72R), KRAS (p.G12C), PIK3CA (p.E545K).*
	PAT5	*APC (p.R876TER, p.M1413TER), TP53 (p.R273H, p.P72R), NRAS (p.Q61K)*	*APC (p.R876TER, p.M1413TER), TP53 (p.R273H, p.P72R), NRAS (p.Q61K), PIK3CA (p.M1043I), SMAD4 (p.Q256TER).*
	PAT6	*APC (p.H298TER, p.K581TER), TP53 (p.P72R).*	*APC (p.H298TER, p.K581TER), TP53 (p.P72R), PIK3CA (p.R88Q), BRAF (p.P403L).*

MT: Metastatic Tumor; PAT: Patient; PT: Primary Tumor. * See methods for TP53 *p*.P72R.

**Table 4:** Distribution of T-cell subsets densities (cells/mm^2^) in primary and metastatic tumors.

Patients	Primary Tumors								Metastatic Tumors							
	CD3+		CD8+		Foxp3		GrzB		CD3+		CD8+		Foxp3		GrzB	
	TC	IM	TC	IM	TC	IM	TC	IM	TC	IM	TC	IM	TC	IM	TC	IM
PAT1	600 ± 15.5	2000 ± 21.2	50 ± 5.5	150 ± 11.1	67 ± 5.4	40 ± 4.2	32 ± 2.7	45 ± 3.3	200 ± 9.9	1500 ± 24.9	50 ± 2.3	120 ± 8.7	20 ± 2.1	40 ± 3.6	74 ± 6.8	30 ± 2.2
PAT2	589 ± 13.2	305 ± 14.5	50 ± 10.6	150 ± 12.2	48 ± 4.8.	9 ± 0.8	30 ± 2.2	40 ± 3.2	200 ± 11.6	500 ± 21.4	70 ± 5.8	100 ± 12.7	50 ± 5.1	45 ± 2.9	44 ± 3.3	25 ± 3.2
PAT3	400 ± 9.5	500 ± 23.2	80 ± 6.2	200 ± 8.4	40 ± 3.3	40 ± 3.9	25 ± 2.1	30 ± 2.6	530 ± 19.2	2015 ± 23.8	78 ± 6.8	100 ± 8.8	10 ± 0.7	10 ± 1.4	58 ± 3.6	45 ± 4.8
PAT4	800 ± 17.4	500 ± 20.1	60 ± 5.4	700 ± 22.5	55 ± 4.2	22 ± 4.1	22 ± 1.6	27 ± 1.9	200 ± 12.1	2014 ± 20.7	154 ± 10.2	110 ± 11.6	10 ± 0.8	35 ± 2.6	33 ± 2.2	50 ± 3.7
PAT5	820 ± 19.7	1500 ± 17.2	600 ± 26.2	500 ± 18.4	30 ± 3.3	20 ± 2.9	120 ± 13.3	110 ± 13.9	130 ± 8.2	1610 ± 19.8	30 ± 2.8	60 ± 6.3	11 ± 0.7	15 ± 1.4	24 ± 2.6	35 ± 2.7
PAT6	2000 ± 23.4	1040 ± 16.1	100 ± 7.4	400 ± 12.3	30 ± 4.2	30 ± 3.3	25 ± 1.6	42 ± 2.9	310 ± 12.1	3000 ± 20.7	100 ± 10.3	530 ± 21.6	28 ± 1.7	30 ± 2.6	26 ± 2.4	42 ± 3.3

Foxp3: forkhead box P3; GrzB: Granzyme B; IM: Invasive Margins; PAT: Patient; TC: Tumor Core.

## Supplementary Materials

The following are available online at https://www.mdpi.com/2072-6694/12/10/3073/s1, Figure S1: Inclusion and exclusion criteria flow-chart for patients’ selection, Figure S2: Clinical courses of group A and group B patients, Figure S3: Representative model showing divergent mutational and immunologic dynamics between oligo- and poly-metastatic colorectal cancer; Table S1: List of genes mutations gained by liver metastases (all coding variants), Table S2: Results of studies reporting mutational evolution of matched primary/secondary lesions in poly-metastatic CRC.
