# Supplementary material for: Evolution of Mutational Landscape and Tumor Immune-Microenvironment in Liver Oligo-Metastatic Colorectal Cancer
Source: Cancers (Basel). 2020 Oct 21;12(10):3073. doi: 10.3390/cancers12103073 (PMC7589866; doi:10.3390/cancers12103073)
Supplement: Supplementary file 1 [file cancers-12-03073-s001.pdf]

cancers

Article

Evolution of Mutational Landscape and Tumor Immune-Microenvironment in Liver Oligo-Metastatic Colorectal Cancer

Alessandro Ottaiano, Michele Caraglia, Annabella Di Mauro, Gerardo Botti, Angela Lombardi, Jerome Galon, Amalia Luce, Luigi D'Amore, Francesco Perri, Mariachiara Santorsola, Fabienne Hermitte, Giovanni Savarese, Fabiana Tatangelo, Vincenza Granata, Francesco Izzo, Andrea Belli, Stefania Scala, Paolo Delrio, Luisa Circelli and Guglielmo Nasti

Supplementary Materials:

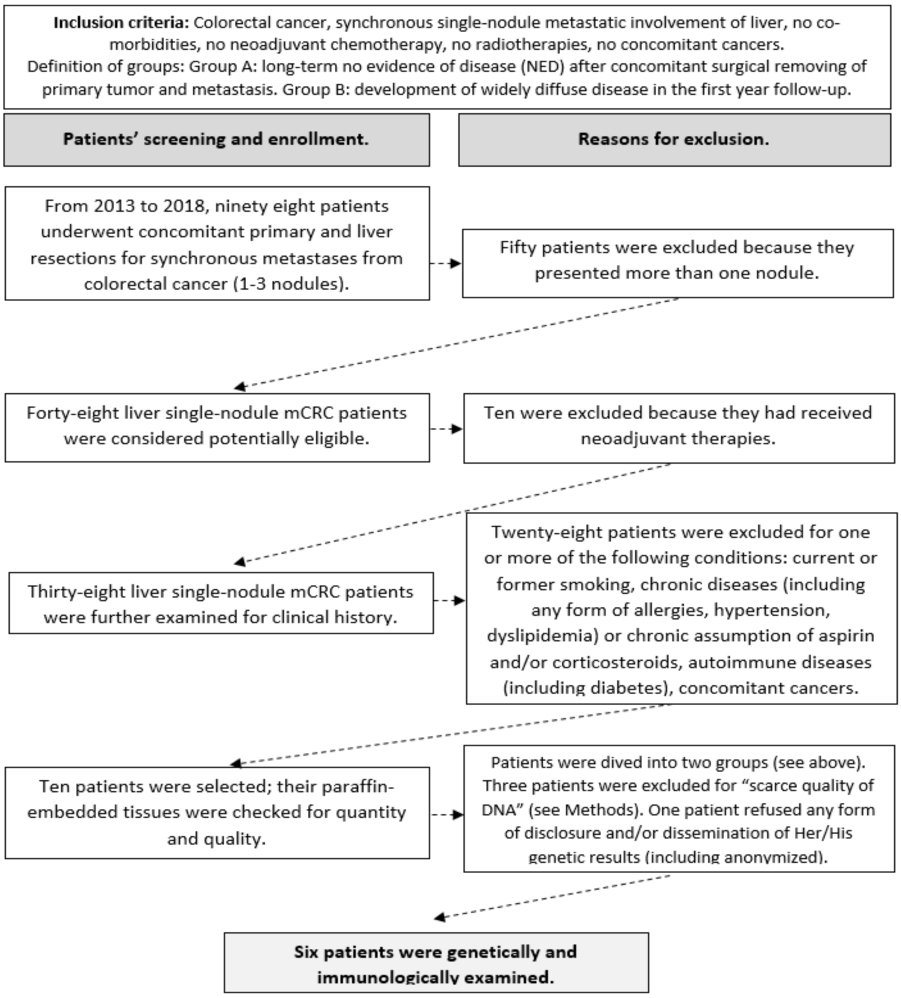

Figure S1. Inclusion and exclusion criteria flow-chart for patients' selection.

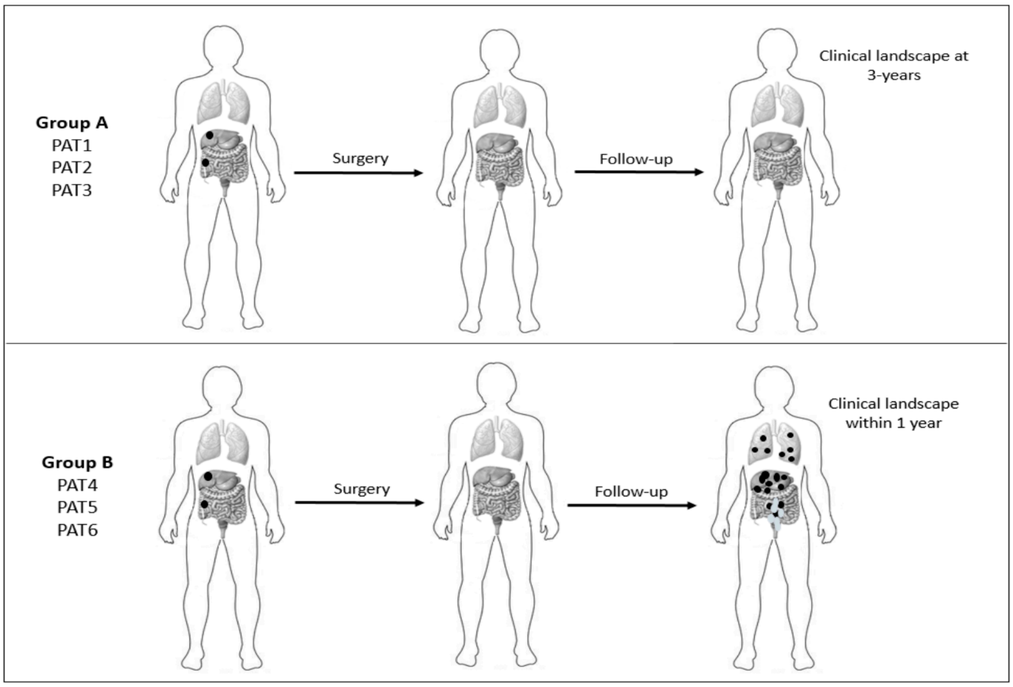

Figure S2. Clinical courses of group A and group B patients.

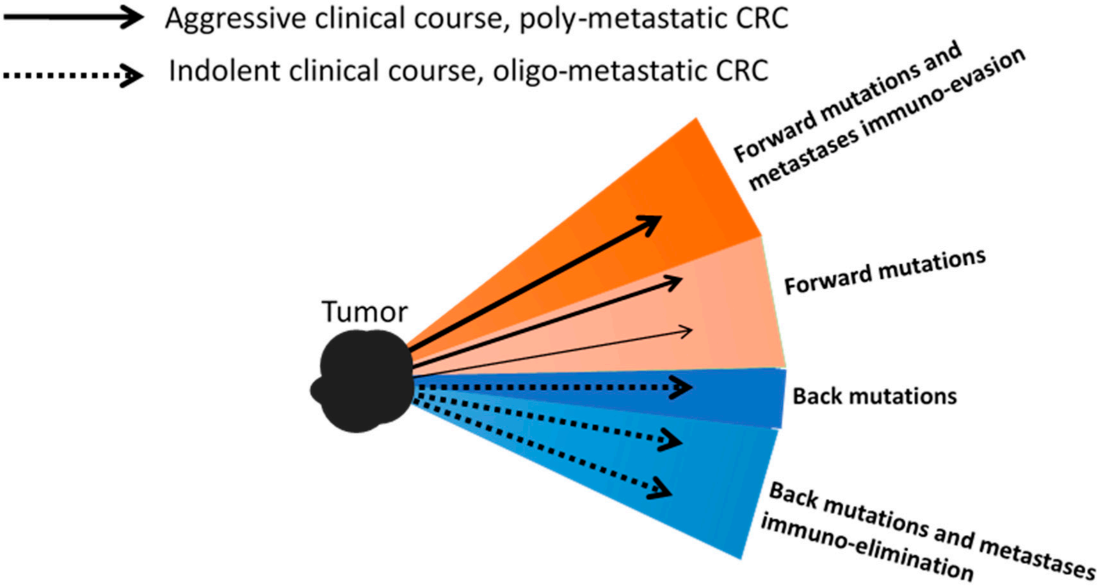

Figure S3. Representative model showing divergent mutational and immunologic dynamics between oligo- and poly-metastatic colorectal cancer.

Table S1. List of genes mutations gained by liver metastases (all coding variants).

Patient	Mutated genes		Role	AMP/ACMG prioritization	ClinVar ID
			Group A		
PAT1	*PRKDC*	p.Gln281His8:g.48855892C>A	DNA repair and recombination	Tier 4	NR
PAT2	*ACVR1B*	12:g.52374752G>T (SAV)	Proliferation, differentiation	Tier 4	NR
	ALK	p.Ala96Thr2:g.30143240C>T	Proliferation, differentiation	Tier 3	451139
	CREBBP	p.Pro1983Thr16:g.3779101G>T	Transcriptional coactivation	Tier 3	NR
	DNMT3B	p.Gly76Arg20:g.31372585G>A	Epigenetic modifications	Tier 3	NR
	FAS	p.Cys135ValfsTer5210:g.90768707CT>C	Programmed cell death	Tier 3	NR
	FGF2	p.Asp179ArgfsTer54:g.123748460A>AC	Proliferation, angiogenesis	Tier 4	NR
	IFNGR1	p.Pro431Ser6:g.137519347G>A	Immune response	Tier 4	NR
	IRF2	p.Gly270Cys4:g.185310154C>A	Transcription factor	Tier 4	NR
	KEL	p.Arg180His7:g.142655047C>T	Zinc endopeptidase	Tier 4	NR
	MDC1	p.Arg1933Gln6:g.30670948C>T	DNA repair	Tier 4	NR
	INSR	p.Ala2Gly19:g.7293898G>C	Glucose homeostasis	Tier 3	190228
	NUP93	p.Gln813Ter16:g.56878498C>T	Programmed cell death	Tier 4	NR
	SOX17	p.Arg142Cys8:g.55371734C>T	Transcription factor	Tier 3	NR
PAT3	*APC*	p.Glu1309AspfsTer45:g.112175211TAAAAG>T	Tumor suppressor gene	Tier 3	15855
	HGF	p.Pro325Thr7:g.81358988G>T	Proliferation, differentiation, cell motility	Tier 3	NR
	MLLT3	p.Ser390_Ser391del9:g.20365693AAGCTGG>A	Transcription factor	Tier 3	NR
	ESR1	p.Lys180Arg 6:g.152163818A>G	Transcription factor	Tier 3	NR
			Group B		
PAT4	*APC*	p.Glu477Ter 5:g.112162825G>T	Tumor suppressor gene	Tier 3	NR
	APC	p.Glu1494GlyfsTer20 5:g.112175769C>CA	Tumor suppressor gene	Tier 3	NR
	APC	p.Glu1494Lys 5:g.112175771G>A	Tumor suppressor gene	Tier 3	454791
	FBXW7	p.Arg689Trp 4:g.153244092G>A	Proliferation	Tier 3	NR
	H3F3C	p.Arg18Gly12:g.31945049G>C	Proliferation	Tier 4	NR
	INSR	p.Arg399Gln 19:g.7172373C>T	Glucose homeostasis	Tier 3	NR
	KRAS	p.Gly12Cys 12:g.25398285C>A	Proliferation	Tier 2	27617
	PIK3CA	p.Glu545Lys 3:g.178936091G>A	Proliferation	Tier 2	28694
	ROS1	p.Lys1766Tyr 6:g.117650559TTTT>TATA	Proliferation, differentiation	Tier 3	NR
PAT5	*PIK3CA*	p.Met1043Ile3:g.178952074G>T	Proliferation	Tier 2	173901

	SMAD4	p.Gln256Ter18:g.48584593C>T	Tumor suppressor gene	Tier 3	NR
PAT6	*ARID1A*	p.Pro1619GlnfsTer71:g.27101569TC>T	Transcription regulation	Tier 3	NR
	B2M	p.Ser16AlafsTer2715:g.45003785CTCTT>C	Immune response	Tier 4	NR
	BRAF	p.Pro403LeufsTer87:g.140482926AG>A	Proliferation , differentiation	Tier 3	NR
	CDK12	p.Gly1461AlafsTer3817:g.37687471TG>T	Proliferation	Tier 3	NR
	DNMT3B	p.Leu454SerfsTer13620:g.31384650AG>A	Epigenetic modifications	Tier 3	138801
	EPHA3	p.Met726CysfsTer53:g.89480334TG>T	Proliferation, differentiation	Tier 3	NR
	ERBB3	p.Arg1080ValfsTer2212:g.56494876GC>G	Proliferation, differentiation	Tier 3	NR
	FGF2	p.Ala212Val4:g.123797533C>T	Proliferation, angiogenesis	Tier 4	NR
	GRM3	p.Arg59Ter7:g.86394636C>T	Proliferation	Tier 4	NR
	JAK2	p.Leu309Arg9:g.5054874T>G	Immune response	Tier 3	NR
	LAMP1	p.Leu276ArgfsTer2113:g.113974735CTG>C	Migration and angiogenesis	Tier 4	NR
	NAB2	p.Pro211LeufsTer5812:g.57485449TC>T	Transcription regulation	Tier 4	NR
	NRG1	p.Asp202Asn8:g.31498104G>A	Proliferation, differentiation	Tier 4	NR
	NOTCH3	p.Gly2035ValfsTer5019:g.15272336CG>C	Proliferation, differentiation	Tier 3	NR
	PIK3CA	p.Arg88Gln 3:g.178916876G>A	Proliferation	Tier 3	362928

SAV: splice acceptor variant

Table S2. Results of studies reporting mutational evolution of matched primary/secondary lesions in poly-metastatic CRC. *when the data were not clearly reported they were derived from Venn Diagrams or descriptive tables.

**Author**	**Year**	**No. of Paired Samples (PT/MT)**	**Patients' Characteristics at Diagnosis**	**Site of Metastases**	**NGS Platform**	**Genetic Sharing PT/MT (Global Concordance)**	**Four Most Frequent and Shared Mutations**	**Unshared Altered Genes in PT (Found in Primary only)**	**Unshared Altered Genes in MT (Found in Metastasis only)**	**TMB**
Brannon AR et al.	2014	69	Four pts stage II, 3 stage III, 62 stage IV. Seventy-five percent of metastases were synchronous. Allowed multiple chemotherapeutic lines. Thirty pts were chemonaive.	Liver (only two ovary).	Illumina, HiSeq 2000.	79%	APC, TP53, KRAS, PI3KCA were the most frequently mutated genes and most highly concordant between PT and MT (concordance of KRAS, NRAS, or BRAF was 100%).	ALK, APC, ASXL1, BAP1, CARD11, CBL, CEBPA, EPHA3, EPHA6, EPHA7, EPHB1, ERBB2, ERBB4, FLT1, FOXL2, GRIN2A, KDM6A, KDR, LGR6, MDM4, MITF, NFKB2, NOTCH3, PBRM1, PDGFRB, PIK3CA, PIK3CD, PIK3CG, SMAD4, STK11, TET1, TP53, TSHR.	APC, AR, ATM, ATRX, BCL6, BRCA2, EGFR, EPHA5, EPHA6, EPHB1, ERBB4, FAS, FH, FLT1, MAP2K1, MAP2K1, NF1, NFE2L2, NOTCH1, NTRK3, PIK3C2G, PIK3CA, PIK3CA, PIK3CD, PIK3CG, PIK3R1, PREX2, PTEN, PTPRS, REL, REL, SMAD4, SMAD4, SUFU, TBK1, TET1, TET2, TGFBR2, TP53, TSHR.	Not reported
Lee SY et al.	2014	15	Stage IV. 6 pts had single liver metastasis. Allowed multiple chemotherapeutic lines.	Liver.	Illumina, HiSeq 2000.	*Mutational concordance showed only for each genes: APC: 100% TP53: 70% KRAS: 100% SMAD4: 75%.	APC, TP53, KRAS, SMAD4. APC and KRAS mutations were ever concordant between PT and MT.	BRAF, CTNNB1, FBXW7, PIK3R1, TP53, SOX9.	ATR, BRAF, CDC42BPG, FBXW7, FLT4, KDR, PI3KCG, RB1, SMAD4, SOX9.	Not reported

Kim R et al.	2015	19		Twelve pts were stage IV, 7 pts stage III. Data on treatments not reported.	Liver, lungs, lymphnodes, ovary.	Illumina, GAIIX.	93.5%	APC and TP53 found concordant in 10/19 pairs. KRAS ever concordant (9/19 pts). PI3K ever concordant (3/19 pts).	ABCA3, ADAMTS20, APC, BRCA2, CX3CR1, DGKB, ERBB4, HSP90AB1, ITGA10, ITGAL, JAK1, LRP1B, MACF1, MAP3K, MAGI2, MARK1, NTRK2, PARP14, PIK3CG, RASA1, ROBO1, SMAD2, SMAD3, SMAD4, TEX14, TNKS, TP53, TTN, WNT2, ZNF217, ZNF831.	ADAMTS18, ADAMTS20, ADCY1, APC, BCL9, CASC5, CHD5, CIC, COL7A1, CSMD3, EPHA5, ETV4, FANCG, FBXW7, GPC5, HERC1, KIAA1409, KNTC1, MACF1, MAPK10, MAST4, MGA, MGMTk, MMP2, MPL, MUC16, NOS1 , PCM1, PPM1H, PREX1, PRKCZ, PTPN13, PTPRC, PTPRD, RASA1, RB1CC1, ROBO1, RPS6KB2, SIRT6, SNX13, STIM1, TACR3, TCF12, TCF3, TOP2B, TOPBP1, TP53, TPO, TRAF4, TTN, VRTN, WNT2.	Not reported
Vignot S et al.	2015	13		Stage IV. Six synchronous metastases, 7 methacronous. Patients received chemotherapy and/or radiotherapy (one pt) before surgery.	Multiple sites. Only local (1 pt), only peritoneum (1 pt).	Illumina, HiSeq 2000.	78%	APC, TP53, KRAS, and SMAD4 were the most frequent mutated genes. Mutated APC had a concordance of 100%.	ALK, BRCA2, GNAS, NF1, RICTOR, STK11, TNKS.	BRCA2, CDH2, CDKN2A, EPHB1, GLUCY1A2, PI3KCG, RB1, RET, SMO.	Not reported
Kovaleva V et al.	2016	14		Stage IV. Synchronous and/or metachronous liver and/or lung	Liver and lungs.	TruSeq Amplicon Cancer PanelTM,	*From 0 to 100% (median 8.5%).	TP53, APC, KRAS, SMAD4.	ABL1, ATM, BRAF, EGFR, ERBB4, FBXW7, FGFR3, GNA11,	ABL1, AKT1, ALK, ATM, BRAF, CDH1, CDKN2A, CSF1R, CTNNB1,	Not reported

	metastases. Allowed multiple chemotherapeutic lines.	MiSeq (Illumina).		GNAQ, HRAS, JAK3, KDR, KIT, MET, NOTCH1, NRAS, PDGFRA, PIK3CA, PTEN, RB1, RET, SMAD4, STK11, TP53, VHL.	EGFR, ERBB2, ERBB4, FBXW7, FGFR2, FGFR3, FLT3, GNA11, GNAQ, GNAS, HNF1A, HRAS, IDH1, JAK3, KDR, KIT, KRAS, MET, MLH1, MPL, NOTCH1, NPM1, NRAS, PDGFRA, PIK3CA, PTEN, PTPN11, RB1, RET, SMAD4, SMARCB1, SMO, SRC, STK11, TP53, VHL.
